# *In Situ* Study of Nanoporosity Evolution during Dealloying AgAu and CoPd by Grazing-Incidence Small-Angle X-ray Scattering

**DOI:** 10.1021/acs.jpcc.1c09592

**Published:** 2022-02-17

**Authors:** Markus Gößler, Elisabeth Hengge, Marco Bogar, Mihaela Albu, Daniel Knez, Heinz Amenitsch, Roland Würschum

**Affiliations:** † Institute of Materials Physics, Graz University of Technology, Petersgasse 16, 8010 Graz, Austria; ‡ CERIC-ERIC C/o Elettra Sincrotrone, S.S. 14 Km 163.5, 34149 Trieste, Italy; § Institute for Inorganic Chemistry, Graz University of Technology, Stremayrgasse 9, 8010 Graz, Austria; ∥ Graz Centre for Electron Microscopy, Steyrergasse 17, 8010 Graz, Austria; ⊥ Institute of Electron Microscopy and Nanoanalysis, Graz University of Technology, Steyrergasse 17, 8010 Graz, Austria

## Abstract

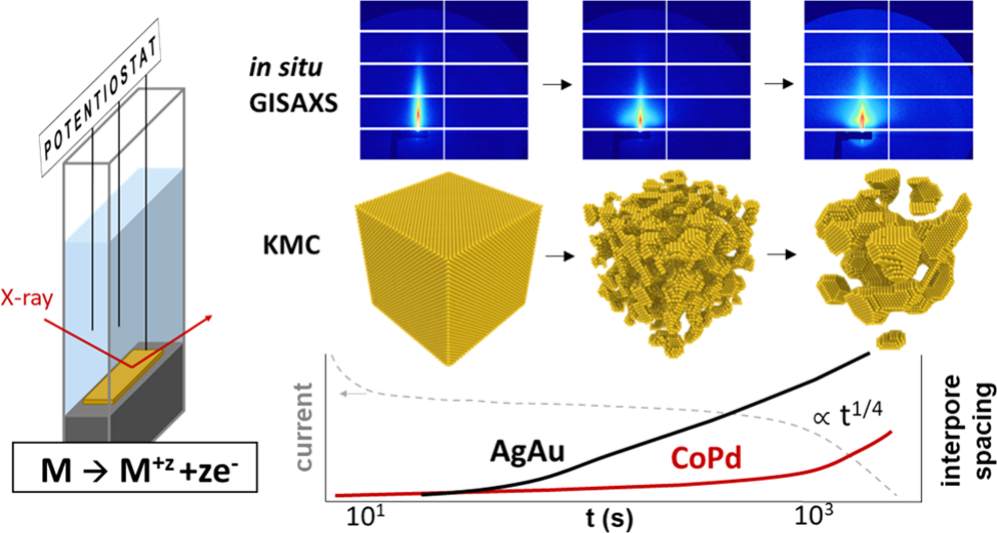

Electrochemical dealloying has become a standard technique to produce nanoporous network structures of various noble metals, exploiting the selective dissolution of one component from an alloy. While achieving nanoporosity during dealloying has been intensively studied for the prime example of nanoporous Au from a AgAu alloy, dealloying from other noble-metal alloys has been rarely investigated in the scientific literature. Here, we study the evolution of nanoporosity in the electrochemical dealloying process for both CoPd and AgAu alloys using a combination of *in situ* grazing-incidence small-angle X-ray scattering (GISAXS), kinetic Monte Carlo (KMC) simulations, and scanning transmission electron microscopy (STEM). When comparing dealloying kinetics, we find a more rapid progression of the dealloying front for CoPd and also a considerably slower coarsening of the nanoporous structure for Pd in relation to Au. We argue that our findings are natural consequences of the effectively higher dealloying potential and the higher interatomic binding energy for the CoPd alloy. Our results corroborate the understanding of electrochemical dealloying on the basis of two rate equations for dissolution and surface diffusion and suggest the general applicability of this dealloying mechanism to binary alloys. The present study contributes to the future tailoring of structural size in nanoporous metals for improved chemical surface activity.

## Introduction

Owing to their high surface-to-volume ratios, nanoporous metals are employed for applications in catalysis^1−3^ and energy.^4−7^ Among various routes for the synthesis of such nanoporous metals,^8^ electrochemical dealloying is a particularly versatile yet simple approach. By selectively dissolving the least noble component from an alloy in an electrolyte under potential control, various bicontinuous nanoporous metals and alloys can be produced.^1,9^ Pore size and composition of dealloyed metals can be controlled via process parameters, such as the type of electrolyte,^10,11^ temperature,^12^ or applied potential.^13−15^

Since Erlebacher devised a microscopic description of the dealloying mechanism based on two simple rate equations for dissolution and surface diffusion,^16^ this model has become the scientific consensus that forms the basic understanding of the dealloying process. Yet several interesting aspects of dealloyed materials are still subject of contemporary research, such as the structural stability and coarsening behavior of the ensuing nanoporous structures^17^ or their mechanical properties.^18^ A broad variety of *in situ* methods have been utilized to study the dealloying process itself, ranging from dilatometry,^19^ resistometry,^20^ magnetometry,^21^ UV–vis spectroscopy,^22^ transmission X-ray microscopy (XTM),^23^ X-ray diffraction (XRD),^24−26^ and Raman spectroscopy^27^ to real-space imaging techniques such as scanning tunneling microscopy^28^ and transmission electron microscopy (TEM).^29^ While microscopic techniques, in general, allow the extraction of size information for the nanoporous structures, they suffer from the disadvantage of averaging over a limited sample area only. Furthermore, definitions of pore size, ligament size, and ligament–ligament distance from images can be inconsistent. A software-assisted evaluation of sizes from electron micrographs improves the reliability of the extracted sizes,^30,31^ although such an approach requires high-quality images. An ideal complement to obtain reliable size information averaged over a larger volume are small-angle scattering methods. Small-angle neutron scattering (SANS)^32^ and small-angle X-ray scattering (SAXS)^33−38^ have been applied to study the dealloying process *in situ*. Although experimental scattering data contain all relevant information about the nanoporous structure, the extraction of sizes characteristic for dealloyed structures has only been shown recently.^37^

Here, we present *in situ* grazing-incidence small-angle X-ray scattering (GISAXS) experiments with enhanced local sensitivity on the surface of the nanoporous materials. In combination with Kinetic Monte Carlo simulations (KMC) and scanning transmission electron microscopy (STEM), we track the evolution of characteristic sizes during electrochemical dealloying of both AgAu and CoPd alloys and apply our findings in the context of current dealloying literature. Ligament–ligament distance and pore size are obtained by fitting GISAXS scattering curves following a model function proposed by Choi and Chen^39^ and first applied to nanoporous metals by Welborn et al.^37^ For comparison of those sizes with KMC and STEM, reciprocal space scattering curves are also calculated from atomic coordinates from the KMC simulation and from STEM using a two-dimensional fast Fourier transformation (2D-FFT) of the images. Ligament–ligament spacing, as the length corresponding to the position of the main scattering peak in reciprocal space, is identified as the proper parameter to describe the size evolution during electrochemical dealloying. Two distinct stages of nanoporosity formation (i.e., dealloying) and a subsequent structural coarsening in the electrolyte are identified in surface area, which is deduced from a fit to GISAXS data following an alternative model after Teubner and Strey.^40^ Characteristic length *d* during coarsening of nanoporous (np)Au from AgAu is found to follow a *d* ∼ *t*^1/4^ law, typical for surface-diffusion-controlled coarsening. A retarded coarsening behavior was found for npPd from CoPd, which is interpreted as a consequence of the higher activation energy for surface diffusion. Our results emphasize the validity of the dealloying mechanism beyond the paramount alloy system of AgAu and provide a general understanding of the electrolytic coarsening of nanoporous metals during dealloying.

## Materials and Methods

### Alloy Preparation

Ag_75_Au_25_ and Co_75_Pd_25_ alloys were prepared via arc melting (Edmund Bühler MAM1) from pure metal precursors (Au, Chempur, 99.95%; Pd, Alfa Aesar, 99.95%; Co, Alfa Aesar, 99.95%; and Ag, Mateck, 99.995%). The AgAu alloy was homogenized at 800 °C for 15 h under Ar atmosphere. While AgAu forms a single-phase over the full compositional range, the Co_75_Pd_25_ alloy was annealed for 1 h at 900 °C in Ar atmosphere and subsequently quenched in water to obtain a single-phase alloy. Both alloys exhibited an fcc crystal structure, as evidenced by X-ray diffraction (XRD) measurements, which are shown in Figures S1 and S2. Both alloys were mechanically rolled to a thickness of 200–250 μm, ground, and polished to a mirror-like finish. After rolling, the AgAu samples were annealed at 650 °C for 1 h in a vacuum furnace. Specimens were cut from the alloy foils to a size of 2 × 12 mm^2^ for *in situ* GISAXS dealloying.

### *In Situ* Grazing-Incidence X-ray Diffraction (GISAXS)

**Figure 1:**
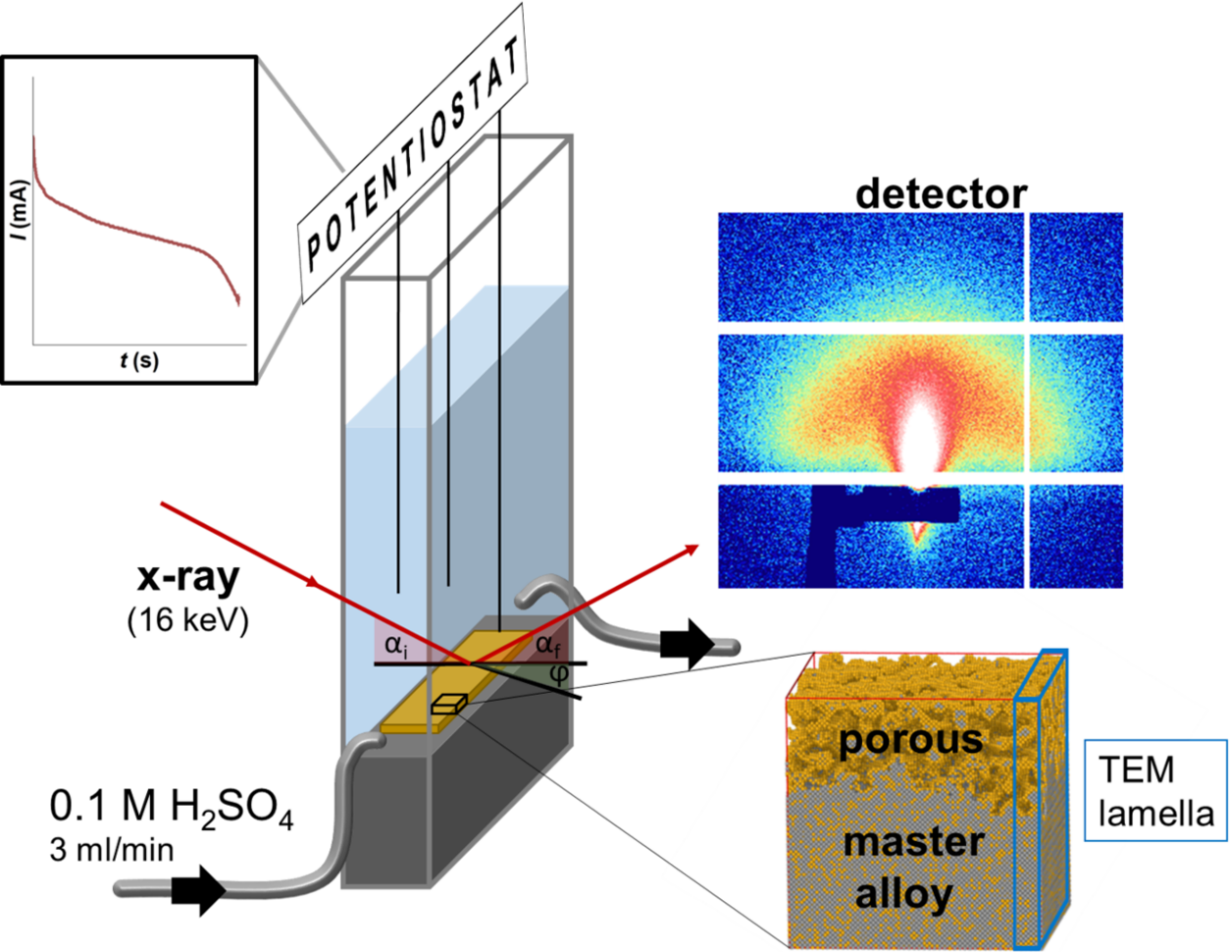
Experimental setup for *in situ* GISAXS during dealloying. Electrochemical dealloying was conducted in a three-electrode electrochemical cell with a continuous electrolyte flow (center left). The corresponding behavior of current over time is schematically depicted in the top left corner. During etching, a detector continuously recorded GISAXS patterns (top right), of which horizontal and radial cuts were extracted for quantitative fitting of size parameters. Alloy specimens were in contact with the electrolyte from the top, which ensured dealloying progress into the sample from top to bottom (bottom right). For STEM imaging, lamellas were cut as indicated in the sketch.

Grazing-incidence X-ray diffraction experiments were conducted at the Austrian SAXS beamline at the ELETTRA synchrotron in Trieste, Italy.^41^ The incoming beam was set at 16 keV (0.77 Å) and directed onto the sample at an incidence angle of α = 0.2°. 2D scattering patterns were recorded by means of a pixel detector (Pilatus3 1 M, Dectris Ltd.) at a distance of 194.63 cm from the sample. The alloy platelets used for dealloying were positioned in a properly designed, 3 mm thin, electrochemical cell optimized for *in situ* GISAXS experiments,^42^ which is schematically shown in Figure 1. The samples were electrically contacted from the top and two Kapton windows allowed X-ray transmission across the cell. Two syringe pumps (Teledyne 500D) were connected to the side plugs of the cell and were used to continuously flow fresh electrolyte above the sample (0.1 M H_2_SO_4_ diluted from a 75% H_2_SO_4_ solution with a Milli-Q water flow rate of 3 mL/min) to remove atoms dissolved from the sample, thus reducing X-ray absorption.

The three-electrode setup in the present case consisted of an alloy platelet as the working electrode, which was fixed and aligned in the cell compartment using setscrews. Connection to a potentiostat (Autolab, PGSTAT204) was established using a Au wire. A homemade Ag/AgCl wire and a Au wire were used as the reference electrode for dealloying CoPd and AgAu, respectively. A coiled Pt wire served as the counter electrode. The electrolyte was 0.1 M H_2_SO_4_ diluted from a 75% H_2_SO_4_ solution with Milli-Q water. For coating the AgCl layer on the Ag/AgCl reference electrodes, Ag wires (0.25 mm, Mateck, 99.995%) were immersed in 3 M KCl solution, when a current density of +1 mA/cm^2^ was applied for 3 min. After every 30 s, the current was reversed for 5 s to obtain a denser coating.

A potentiostatic dealloying potentiostatic dealloying procedure was used for *in situ* measurements, which recorded current as a function of time at a constant potential of *U*_D_ = 0.73 V (*vs* Au) for AgAu and *U*_D_ = 0.55 V (*vs* Ag/AgCl) for CoPd, as schematically shown in Figure 1.

During dealloying, exposure time to the X-ray beam was adjusted as a function of the elapsed time of dealloying as follows: for the first minute of dealloying, the exposure time was set to 0.095 s; during the second minute of dealloying, the exposure time was increased to 0.495 s; and during the third minute of dealloying, the exposure time was then set to 0.995 s. For the following seven minutes of dealloying, the exposure time was set to 8 s and, for the remaining part of the measurement, the exposure time was set to 20 s. The initial, short exposure time was used to record the fast evolution taking place during the very early stages of the process, while its progressive increase allowed us to record the evolution of the sample with a higher signal-to-noise ratio.

The IGOR Pro software (IGOR Pro 7.0.8.1, Wavemetrics) was used for data reduction and fitting. For each sample, the background signal constituted by the experimental cell filled with the electrolyte was subtracted at first. Then, for both samples, analysis was performed from a horizontal cut (in-plane direction) calculated at the height of the Yoneda wings; only for the CoPd sample, an additional radial cut was calculated to reveal the isotropic scattering in transmission, as shown in Figure S3. A first qualitative analysis was carried out by observing the evolution of the calculated scattering correlation length ξ of the horizontal cut.^43,44^ The correlation length ξ was calculated within the range of 0.15–2 nm^–1^ for AgAu and 0.3–2 nm^–1^ for CoPd. For a quantitative analysis, the focus is set on the relative changes of the structural parameters. As a consequence, we have used a simplified analytical model to interpret the in-plane cuts rather than the full application of the distorted wave Born approximation (DWBA).^45^ To monitor the evolution of the nanoporous structure, one-dimensional (1D) scattering patterns of the horizontal cuts were fitted by means of two models: the so-called Choi–Chen model^39^ was used at first, while the Teubner–Strey model^40^ was employed to complete the characterization by including the analysis of the evolution of the surface area, and it was adapted to also model the behavior in the low-*q* region. The Choi–Chen model was developed for studying two-phase systems developing in a three-dimensional environment by means of three main parameters: an interdomain distance (*L̃*), here used to describe average ligament–ligament distance, a coherence length of the local domain order (*R*), here used to describe the average pore radius, and an interfacial length (δ), which takes into account the surface roughness in between the two phases.^39^ From these parameters, the average pore radius was calculated as *D*_P_ = 2(*R* + δ). A power law term was added to take diffuse scattering into account of large structures as well as for the contribution of the specular reflected beam in the in-plane direction. The complete model can be written as
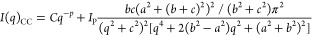
where *a* = 2π/*L̃*, *b* = 1/*R*, and *c* = 1/δ. Although the Choi–Chen model provides a detailed description of a two-phase system, it does not include any information about average surface area. Thus, for monitoring the evolution of the surface area during dealloying, also the Teubner–Strey model was used to fit the experimental data. The Teubner–Strey model is based on an order parameter expansion of the Landau mean free energy density up to second order including two gradient terms. The expansion coefficients *a*_i_ and *c*_j_ are fixed to zero, except *a*_2_ > 0, *c*_1_ < 0, and *c*_2_ > 0.^40^ Indeed, the coefficient *c*_1_ is related to the creation of domain walls between two heterogeneous phases, while the coefficient *c*_2_ concurs in system stabilization. In this way, the term (*a*_2_ + *c*_1_*q*^2^ + *c*_2_*q*^4^)^−1^, for negative values of c_1_, gives rise to a broad structure peak, which is followed by a decaying intensity, proportional to *q*^–4^ in the Porod regime. From the Fourier transformation of the corresponding correlation function (γ(*r*)=*L̃*(2π*r*)^−1^e^–*r*/*R*^ sin(2πr/*L̃*)), two length scales can be found^40^ describing the average size of the pore
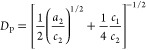
and describing the average ligament–ligament distance.
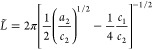
The coefficient *a*_2_ was set to 1. The average surface area was estimated to be proportional to the ratio of forwarded scattering intensity and the coefficient *c*_2_, *I*_TS_/*c*_2_. An additional power law was added to account for large buried aggregates, crack formation, and/or changes of the surface roughness, while the diffuse scattering of the off-specular contribution in the low-*q* region of the horizontal cut was approximated by means of the so-called Guinier–Porod model by Hammouda, *H*(*I*_D_,*R*_g_,*d*,*q*).^46^ Here, the important contributions are *I*_D_ (in the Guinier–Porod model corresponding to the Guinier scaling factor) and the power law *d* representing changes of the surface roughness. Thus, the Teubner–Strey model used for data fitting can be expressed as
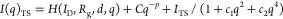
Here, the results of the power laws and the Guinier–Porod model were considered as qualitative measures and not further analyzed. Additionally, the contribution of the Guinier–Porod model was not added when analyzing the radial cut, due to the reduced range of integration in the low-*q* region.

### Scanning Transmission Electron Microscopy (STEM)

For STEM investigation of the nanoporous structures, samples were prepared in the same flow-cell setup as used for *in situ* GISAXS, while all experimental conditions stayed the same. Dealloying was stopped after 1100 s for the AgAu alloy and 4500 s for the CoPd alloy to obtain similar structures as in the late stages of the respective *in situ* dealloying experiments. An alloy backbone was conserved in either case to ensure mechanical stability for the preparation of TEM lamellas, which is schematically shown in Figure 1. Only the topmost layer (5–10 μm) of the nanoporous structures was prepared for STEM investigation utilizing a focused ion beam (FIB) to monitor a similar morphology as in the scattering patterns via GISAXS. The analysis has been performed using the ASTEM probe-corrected Titan^3^ G2 60-300 microscope (Thermo Fisher) operated at 300 kV (beam diameter of 1 Å, convergence angle of 19.6 mrad). Images were acquired by annular and high-angle annular dark-field detectors (ADF and HAADF). The data have been processed by the Gatan Microscopy Suite 3 (GMS).

### Kinetic Monte Carlo Simulation

Kinetic Monte Carlo simulations of the dealloying process were based on rate equations originally proposed by Erlebacher.^16^ The rate equations for surface diffusion, allowed for both atomic species in the alloy, and dissolution of the less noble alloy components (Ag or Co only) are
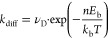
1and
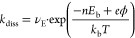
2where *k*_diff_ is the diffusion rate, ν_D_ is the attempt frequency for surface diffusion (set as 10^13^ s^–1^), *n* is the coordination number of the respective lattice site, *E*_b_ is the single-bond binding energy, *k*_diss_ is the dissolution rate, ν_*E*_ is the attempt frequency for dissolution (set as 10^4^ s^–1^), ϕ is the parametrized electrode potential, and *T* is the temperature, with *e* and *k*_b_ being the usual physical constants.

Binding energy *E*_b_ for AgAu was adopted from ref (16), while for the binding energy of CoPd, we used the AgAu value scaled by a factor of 1.2 accounting for differences in melting temperature of the two alloys (*E*_b_ = 0.15 eV for Ag_75_Au_25_, *E*_b_ = 0.18 eV for Co_75_Pd_25_). The electrode potential ϕ for AgAu was set as 1.14 V, which corresponds to the critical dealloying potential of this materials system in the simulation.^16^ Accounting for the higher applied potential for AgAu in the dealloying experiment, as well as the intrinsically different dissolution potentials in the electrochemical series of Ag (+0.8V)^47^ and Co (−0.28 V),^47^ ϕ was fixed to 1.5 V for CoPd. The value of ϕ determines the balance between dissolution and diffusion only on short timescales (τ < 100 s) in the simulation. Simulated real-time τ was calculated incrementally each step following
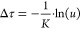
where *K* is the sum of the rates for all processes and *u* ∈ (0, 1] is a uniform random number.

In the post-dealloying coarsening regime, differences in binding energy *E*_b_ are responsible for the time evolution of the structural sizes. The KMC simulation of the dealloying process in this work was implemented in MATLAB. Atomic coordinates of both alloy constituents were exported every 10^4^ steps.

For the calculation of scattering patterns, simulated boxes were randomly rotated using 90°-rotation matrices and replicated on a triclinic lattice with 3 × 4 × 5 sites. This larger structure was created to suppress box-size periodicity in reciprocal space. Lattice constants of the fcc structures were fixed to 3.65 Å for CoPd and 4.07 Å for AgAu. The Crysol^48^ software allowed us to calculate the small-angle X-ray scattering patterns corresponding to Au and Pd nanoporous structures retrieved by means of KMC simulations (maximum order of harmonics: 21, order of Fibonacci grid: 18). Simulated structures were displayed by means of the open-source software OVITO.^49^

## Results

**Figure 2:**
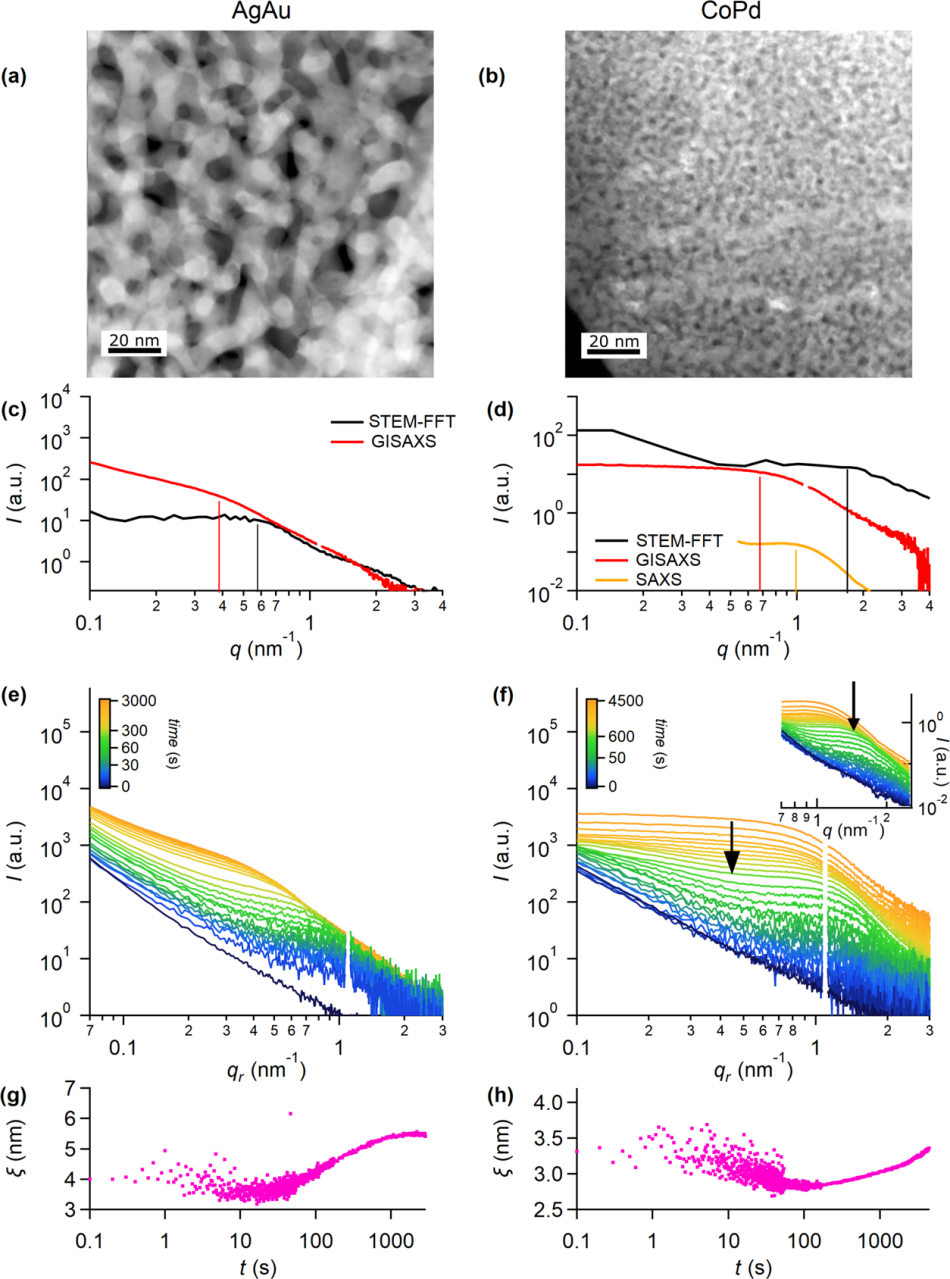
STEM HAADF and small-angle scattering. STEM images after dealloying (a) AgAu and (b) CoPd for 1100 and 4500 s, respectively (scale bar is 20 nm). A comparison of radially averaged 2D fast Fourier transformations calculated from STEM images displayed in black and the corresponding measured GISAXS scattering patterns in red for (c) AgAu and (d) CoPd. Time-resolved in situ GISAXS horizontal cuts recorded for (e) AgAu and (f) CoPd undergoing the respective dealloying procedure. The corresponding time-resolved in situ SAXS radial cuts for CoPd are shown in the inset in (f). The correlation length ξ from experimental data is plotted in (g, h) for AgAu and CoPd, respectively. Note: The transition from scattered data points to a smooth curve is caused by the increasing exposure times to the X-ray beam.

For our comparative study of the dealloying processes for CoPd and AgAu alloys, in a first step, STEM images of dealloyed nanoporous Pd and Au structures are shown in Figure 2a,b in late stages of the dealloying process, i.e., after complete conversion of the alloy into the nanoporous structure in the topmost region (∼100 nm), which is the region investigated experimentally. A significantly coarser structure is observed for nanoporous Au in Figure 2a, compared to nanoporous Pd produced from the analogous experiment in Figure 2b. The disordered and irregular nature of the nanoporous structures apparent in the STEM images illustrates the problem of identifying a representative length on the basis of such images, as one could define ligament width and length, pore size, or interligament spacing. To bypass this problem, we calculated radially averaged 2D fast Fourier transformations (STEM-FFT) of both images, which includes size information in the reciprocal space^50^ (see Figure S4 in the Supporting Information for a schematic representation of the calculation steps). These curves are depicted in Figure 2c for nanoporous Au and 2d for nanoporous Pd. On the basis of peak position *q* in reciprocal space, one could estimate a characteristic length as , which yields ∼10 nm for npAu and ∼3 nm for npPd, which agrees with structural sizes from real-space images. The second sharp peak in the STEM-FFT curve in Figure 2d at lower *q*-values is attributed to the changing contrast in the STEM image in Figure 2b and is considered as an artifact.

As this information in reciprocal space is equivalent to information from small-angle scattering techniques, we directly introduce results from grazing-incidence small-angle X-ray scattering measurements in Figure 2c,d, which were acquired using an equivalent dealloying procedure as for STEM samples. Horizontal cuts from 2D GISAXS patterns for npAu and npPd are depicted in red, alongside the corresponding STEM-FFT curves in black. Intensities for the GISAXS cuts have been rescaled to enable comparability with the STEM-FFT curves. For npPd from CoPd, a radial cut (SAXS geometry) is additionally shown in orange. The shape of GISAXS scattering curves for npAu in Figure 2c closely resembles the STEM-FFT image in the same plot. The knee in the curve, which is indicated with a vertical line, appears at a larger *q*-value for STEM-FFT in black compared to the GISAXS scattering curve in red, which points toward a smaller structural size observed via STEM. Scattering curves for npPd in Figure 2d generally appear at larger *q*-values compared to npAu, which reflects the larger structural sizes for npAu. For npPd, the shift of the scattering curves from STEM-FFT and GISAXS is more apparent, as again seen in the position of the knee, marked by the vertical lines. This larger shift in *q* corresponds to a larger divergence of the structural sizes observed in STEM and GISAXS experiments for npPd. Finer nanoporous structures on the surface compared to the underlying bulk are a known phenomenon for npAu^11,51−53^ and might be responsible for the observed divergence of sizes from scattering patterns and electron micrographs here. STEM images were recorded on the topmost surface layer, where such a finer structure can be expected. GISAXS, on the other hand, has a larger penetration depth of ∼60 nm assuming a classical absorption law due to the rough interface layer, which is enough to observe the larger structural sizes in the bulk nanoporous material.

Finally, Figure 2e,f shows results from our time-resolved *in situ* GISAXS experiments for dealloying of AgAu and CoPd, respectively. Colored traces represent scattering patterns at different points in time, from blue at the start to orange at the end of the measurement. Scattering patterns evolve from featureless power law behavior attributed to surface roughness and partly to the diffuse scattering in the electrolyte, to showing a pronounced scattering peak already after the first seconds of dealloying. With ongoing dealloying, the scattering peaks move toward smaller *q*-values, indicating an increase in the average ligament–ligament distance. The evolution of the size of the system is then reflected in the rising correlation length ξ calculated from the GISAXS patterns, which is shown in Figure 2g for AgAu and in Figure 2h for CoPd.

Moreover, concerning the dealloying of CoPd, a more detailed analysis is required. At the beginning, the GISAXS mode is dominant as the off-specular reflected contribution is very strong in the out-of-plane direction. This can be also observed in Figure S3c in the sharp interface due to the sample horizon. At the end (Figure S3e), there is a more or less isotropic scattering due to the transmission geometry. Over the time course, a significant drop of the specular reflected intensity can be observed. Such a sharp variation is attributed to the mechanical bending of the sample during the experiment induced by the mechanical stress, which is visible also in the variation of the out-of-plane scattering distribution (data not shown). This change of sample geometry caused the incident X-ray beam to pass through the sample and changed the experimental conditions to a transmission experiment. Thus, small-angle scattering analysis needed to be shifted from the initial GISAXS mode to the transmission SAXS mode by performing a radial cut on the 2D scattering pattern, which is shown in the inset in Figure 2f. Although the transition from GISAXS to SAXS does not occur at a precisely defined time, from the evolution of the structural parameter of both techniques, we determined the middle of their overlap regime at a time of 655 s (marked by the black arrow) as the point of transition. This overlap regime was defined within the region in which the dimension of the average ligament–ligament distance retrieved by data fitting was giving roughly the same value.

The effects of this geometrical variation of the CoPd sample are mainly observed in the low-*q* area of the scattering pattern (Figure S5) and were quantified by fitting the two data sets with the Teubner–Strey model; whereas in AgAu, the reduction of the scaling factor *I*_D_ for the Guinier–Porod contribution (off-specular intensity) in the low-*q* region is only slightly decreasing (reduction factor of 10) (Figure S6), in CoPd, there is a quasi-disappearance of this scaling factor *I*_D_ (reduction factor of 400).

**Figure 3:**
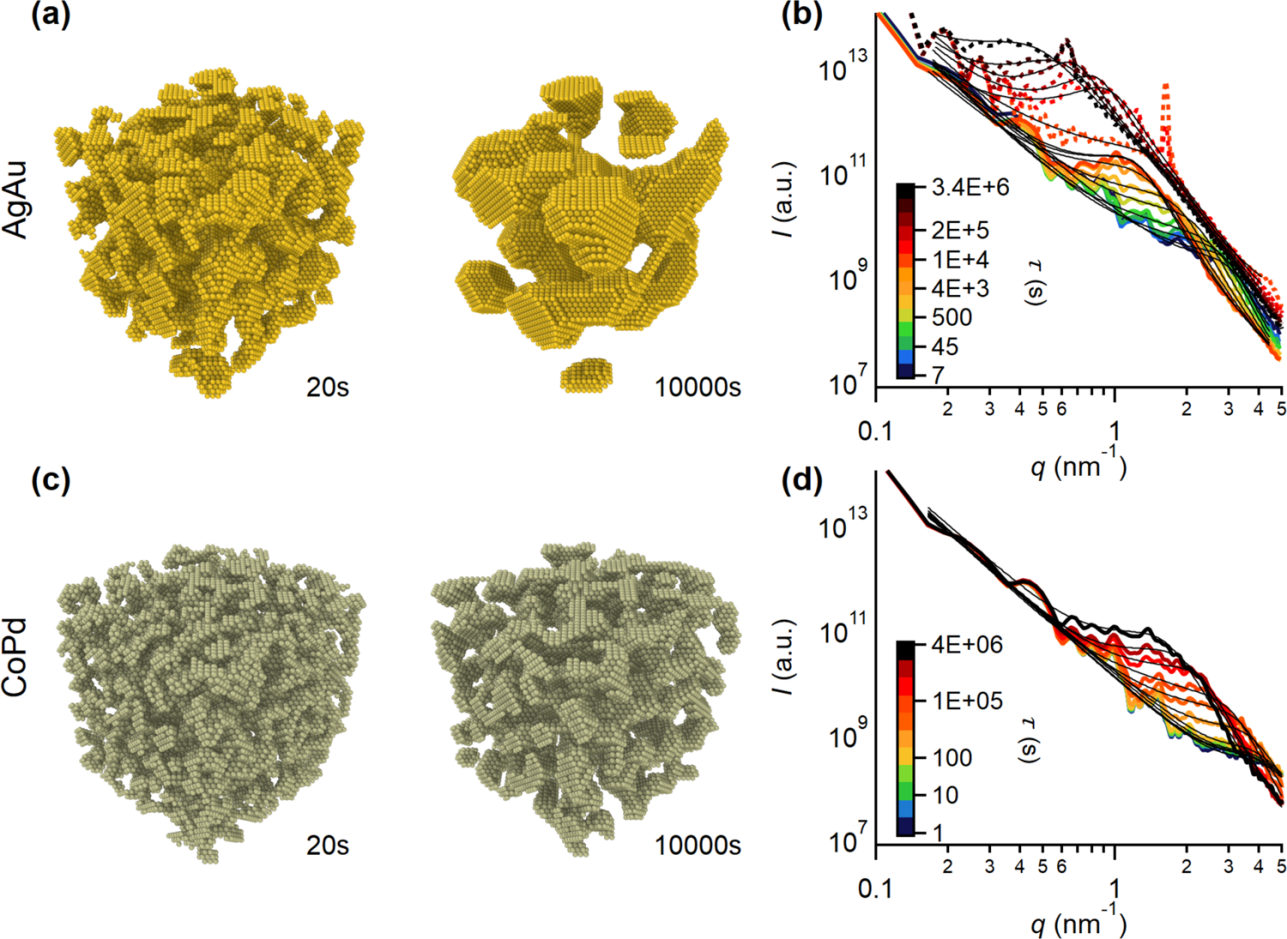
Scattering curves from KMC simulations. Representative real-space structures of a single unit cell with a size of 30 × 30 × 30 atoms from the KMC simulation after τ = 20 s and 10 000 s are shown in (a) for AgAu and (c) for CoPd. Note the finer ligament size (corresponding to a higher *q*-value for the scattering peak) for CoPd. Calculated scattering patterns for (b) AgAu and (d) CoPd from atomic coordinates exported during the KMC simulation. The calculated scattering curves from atomic coordinates for the coarsening of npAu from ref (54) are added as dotted lines. Black lines represent fits to the scattering curves following the Choi–Chen model.^39^ Scattering curves are averaged over several unit cells in a triclinic structure to reduce artifacts related to the finite box size.

For a systematic analysis of our *in situ* GISAXS experiments of AgAu and CoPd dealloying, we performed complementary KMC simulations of the dealloying process for both alloys. Coordinates of atoms were tracked in the course of the simulation and exported every 10^4^ simulation steps. In Figure 3, unit cells from the KMC simulations of 30 × 30 × 30 atoms are shown after a dealloying time of τ = 20 s and 10 000 s for AgAu (a) and CoPd (c). Apparently disconnected regions in the box are artifacts arising from the periodic boundary conditions in two dimensions. Dealloyed nanoporous structures appear coarser for npAu compared to npPd after both 20 and 10 000 s of dealloying, which is in line with results from STEM and GISAXS in Figure 1. For both npAu and npPd, a coarsening with progressing time is also apparent. For a direct comparison with GISAXS data, scattering curves from STEM micrographs for different dealloying times were calculated from the atomic coordinates using the Crysol software (see the Materials and Methods Section), which are displayed in Figure 3b,d. A pronounced peak is visible for both npAu and npPd, which shifts toward lower *q*-values with increasing time. Simulated scattering patterns accurately reflect the behavior from the GISAXS experiments in Figure 2e,f. Both scattering curves from experiment and simulation were modeled to extract quantitative size parameters, characteristic for the nanoporous structures, with the fits according to the Choi–Chen model^39^ drawn as black lines in Figure 3b,d. The temporal evolution of characteristic size parameters during dealloying extracted from this model and their interpretation are the subject of the next section.

## Discussion

### Characteristic Length Scale during Dealloying

Nanoporous metals prepared via electrochemical dealloying bear morphological resemblance to two-phase systems obtained via spinodal composition. Both represent examples for nonperiodic, bicontinuous structures of two intertwined phases, generated via a phase separation process. Nowadays, such structures are successfully modeled using Gaussian random fields, where the two different phases are separated using a level cut.^55^ Random fields are generated as a superposition of standing waves of random phase and direction but with only a single underlying wavelength λ. For nanoporous structures, this wavelength λ has been used to define a characteristic spacing *L̃*(55,56) between centers of neighboring ligaments via the first maximum in the correlation function. In the KMC simulation study of Li et al.^56^ on the coarsening behavior of nanoporous gold, this characteristic spacing *L̃* was shown to be the size parameter most suitable for studying the size evolution during coarsening, compared to apparent ligament sizes deduced from electron micrographs or inverse surface area. For the present study on the size evolution during dealloying, we use small-angle X-ray scattering to uncover the underlying wavelength of the nanoporous structure. Through model fits to reciprocal space data from the experiment and simulated scattering curves from KMC simulations, we probe the size parameter *L̃* consistently for both methods.

Size information at a given point of time *t* in the experiment or τ in the KMC simulation is extracted from fits to the experimental scattering curves in Figure 2e,f and the simulated scattering curves in Figure 3b,d, as described in the Materials and Methods Section. The Choi–Chen model^39^ enables a robust fitting of scattering curves from nanoporous materials with a broad scattering peak using three underlying length scales, first applied to dealloyed nanoporous materials by Welborn et al.^37^

Two of these length scales are of special relevance for the dealloying process: (1) The characteristic ligament–ligament spacing *L̃*, as introduced above. As *L̃* is connected to an underlying wavelength, it can be considered as a measure for periodicity in the nanoporous structure. This quantity *L̃* scales inversely with the peak position in the scattering curve. (2) A ligament or pore diameter *D*_p_. In terms of scattering, this is interpreted as a measure for the spatial decay of electron density fluctuations.^37^

**Figure 4:**
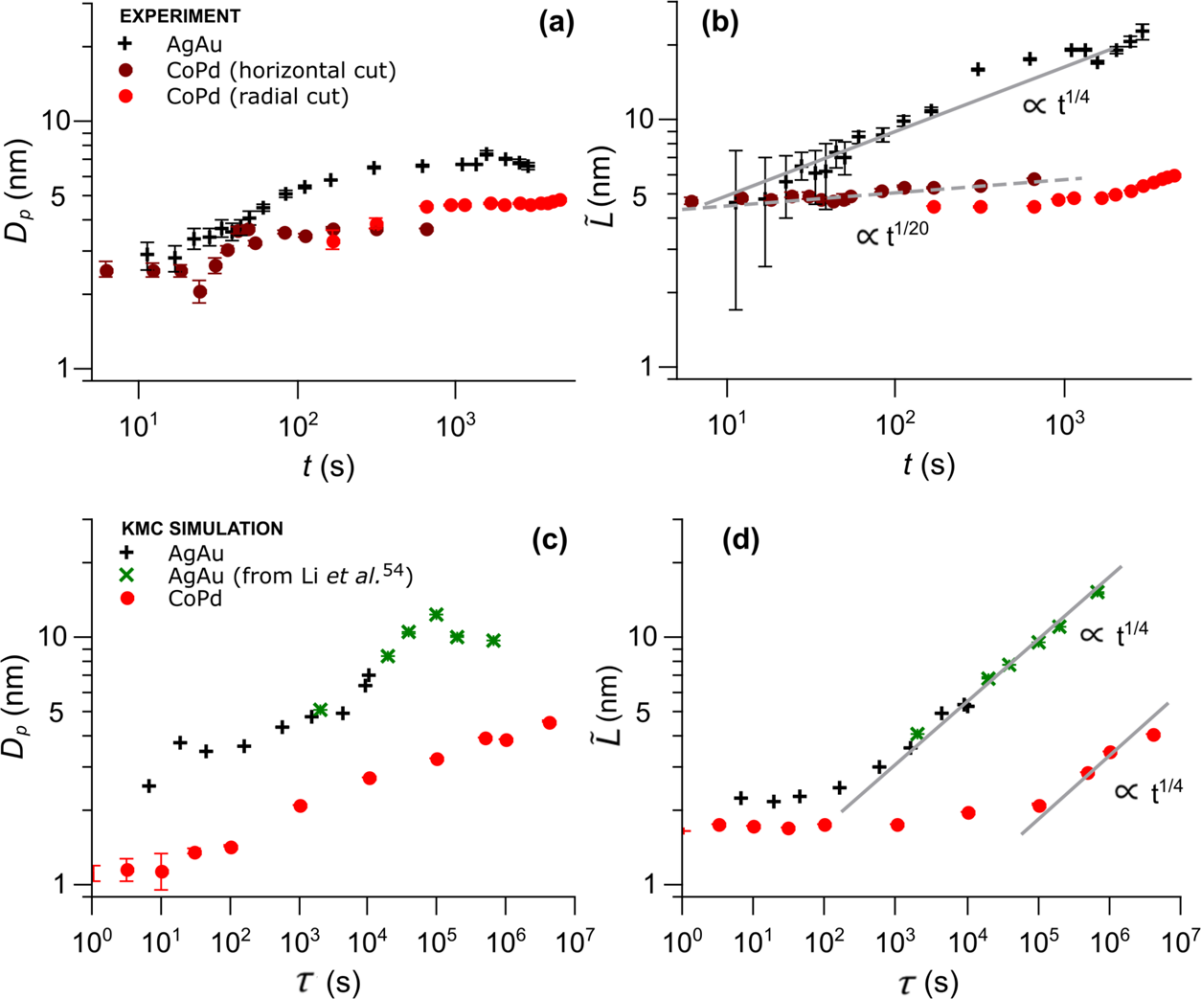
Evolution of structural sizes during dealloying. Pore size *D*_P_ and interpore spacing *L̃* as a function of etching time *t* from experiment (a, b) and as a function of simulated time τ from KMC simulations (c, d) in double-logarithmic representation. Note the different *x*-axis scaling for the lower and upper graphs. Size parameters are obtained via fitting horizontal cuts of the GISAXS patterns in Figure 2 and calculated scattering curves from KMC simulation in Figure 3 using the Choi–Chen model.^39^ For CoPd, both radial and horizontal cuts of the GISAXS patterns were fitted. The same fitting procedure was used on calculated scattering curves from literature KMC data^54^ for the temperature-driven coarsening of nanoporous Au at 900 K, which are included in (c, d) as a reference with a shifted timescale (green crosses). Data points for AgAu (CoPd) are shown as black crosses (red circles) in all subplots. Continuous lines correspond to a slope of 0.25, which is characteristic for surface diffusion-driven coarsening (*d* ∼ *t*^1/4^).

In our comparative study for dealloying of Au and Pd, the characteristic parameters *L̃* and the additional parameter *D*_p_ from the Choi–Chen model are extracted in Figure 4 for both elements from experimental GISAXS data (a,b) and from the calculated scattering curves from the KMC simulation (c,d) as a function of experimental time *t* and simulated time τ, respectively.

Our approach of extracting the ligament–ligament diameter *L̃* and ligament/pore size *D*_p_ from both experimental and simulated scattering curves from KMC simulations enables direct comparability of all relevant length parameters between experiment and simulation. In contrast to the previously used calculated *L̃* from specific surface area and porosity,^56^ the direct fitting of simulated scattering curves offers additional advantages if deviations from a self-similar structure evolution occur, i.e, if not only the scale but also the morphology of the structure changes. The evolution of the characteristic ligament–ligament distance *L̃* and pore/ligament size *D*_P_ in the dealloying process is discussed in the following.

### Dealloying Kinetics

First, we consider the experimental length scales for AgAu and CoPd in Figure 4a. In the double-logarithmic representation, *D*_P_ increases monotonously up to a plateau at *D*_P_ = 7 nm for AgAu and a plateau at *D*_P_ = 5 nm for CoPd. The ligament or pore size *D*_P_ is systematically larger for AgAu compared to CoPd by about 2 nm for all data points. The behavior of the ligament–ligament spacing *L̃* as a function of time *t* in Figure 4b shows a monotonous increase for all data points for both AgAu and CoPd, with a noticeably larger slope for AgAu. The gray line in Figure 4b represents an increase of *L̃* following a *t*^1/4^ law, which points toward a surface-diffusion-driven coarsening.^57^ Data points for the dealloying of AgAu roughly follow this *t*^1/4^-behavior, which is in line with previous studies on the coarsening of npAu.^12,38,58^ The ligament–ligament spacing *L̃* for CoPd, on the other hand, shows a considerably smaller slope, following a coarsening behavior with a much smaller coarsening time exponent of about *t*^1/20^ (dashed gray). A small jump is apparent in the data points for CoPd at the transition from GISAXS fitting of horizontal cuts to the SAXS fitting of radial cuts, which arises due to systematic scaling differences for the two fitting methods as GISAXS probes the in-plane component and SAXS probes the radially averaged components.

In Figure 4c,d, the same parameters *D*_P_ and *L̃* were obtained for scattering patterns calculated from KMC simulation data. It should be noted that experimental timescales *t* in Figure 4a,b and timescales for the simulation τ in Figure 4c,d cannot be directly compared, due to an influence of the box size and rate parameters on the simulated time τ from the KMC simulation. Nonetheless, all data points for CoPd and AgAu in the respective subplots are fully comparable, regardless of the *x*-axis being experimental time *t* or simulated time τ. For further comparison with literature data, KMC simulations for the thermal coarsening of nanoporous Au with a porosity φ = 0.35 at 900 K have been evaluated. Data for these simulations have been previously published in the study of Li et al.,^54^ with atomic coordinates at various coarsening stages available online. In analogy to our in-house KMC simulation of the dealloying process, atomic coordinates from the reference KMC coarsening simulation^54^ were used to calculate scattering patterns, which in turn were fitted using the Choi–Chen model. Times from the reference were rescaled to match size parameters from the later stages of the KMC dealloying simulation and enable a comparison of electrolytic and thermal coarsening.

The ligament or pore size *D*_P_ for both AgAu and CoPd in Figure 4c shows similar features compared to the experimental values of *D*_P_ in Figure 4a. Systematically larger values are again observed for the AgAu dealloying simulation compared to CoPd, while both are in decent agreement with the values for *D*_P_ obtained from the experiment in Figure 4a. The plateau at higher dealloying times in Figure 4a, however, does not exist for pore sizes extracted from the KMC simulations in Figure 4c. Data points from the high-temperature coarsening KMC reference^54^ in green perfectly match the slope from the dealloying KMC simulation at larger times in black, indicating that the same mechanism is responsible for both high-temperature and electrolytic coarsening. The ligament–ligament distance *L̃*, as the characteristic parameter for the size evolution, is depicted in Figure 4d for the KMC simulation of AgAu and CoPd. The curves show the same trend as values for *L̃* from the experiment in Figure 4b. After an initial period of constant *L̃*, a linear increase is observed for larger simulated times τ. This is in excellent agreement with the theoretical prediction of a surface-diffusion-controlled growth with a coarsening exponent of *n* = 4 (*L̃* ∼ *t*^1/4^), represented by the gray lines. Values for *L̃* from the simulation are generally lower compared to the experimental values in Figure 4b, possibly connected to an overestimation of the binding energy parameter in the KMC simulation.

An unexpected new finding is the retarded coarsening behavior of npPd compared to npAu, which is evident in the size parameter *L̃* from both the GISAXS experiment and the KMC simulation in Figure 4b,d. In the following, we argue that this is a natural consequence of the higher binding energy for Pd compared to Au and interpret it as a period of dominant structural faceting.

Studies on the coarsening of nanoporous Au^12,38,58^ generally report a decent agreement with a kinetic *t*^1/4^ -scaling law behavior, typical for a surface-diffusion-driven process. In contrast, Son et al.^59^ observed a considerably slower thermal coarsening behavior of npAu using a combined experimental and KMC simulation approach. They found that the typical coarsening exponent for surface diffusion *n* = 4 describes the temporal evolution during annealing only at sufficiently large temperatures. For lower annealing temperatures of *T* = 450 °C, a much larger coarsening exponent in the order of *n* ∼ 12.5 (*L̃* ∼ *t*^0.08^) was extracted, which indicates a retarded coarsening behavior similar to our observation for the electrolytic coarsening of npPd.

In the recent KMC simulation study by Li et al.^56^ on the coarsening behavior of npAu, a slower coarsening behavior has been found at a temperature of *T* = 900 K compared to a higher annealing temperature of *T* = 1800 K, which has been ascribed to a more pronounced stage of faceting at lower *T*. They argue that a single activation energy can account for both low- and high-temperature coarsening kinetics, as both coarsening curves coincide when rescaling the time axis with a constant factor (corresponding to a shift on the logarithmic *x*-axis). An analogous argument is advanced in the present study for electrolytic coarsening, as the evolution of the characteristic size parameter *L̃* for AgAu and CoPd in Figure 4b,d also follows the same behavior on different timescales. In Figure 4b,d, a period of retarded coarsening is observed for npPd (*L̃* ∼ *t*^1/20^), with a stronger increase in characteristic size commencing only at larger times. Simulated scattering data indicate a transition to a typical *L̃* ∼ *t*^1/4^ behavior for large enough dealloying times. In analogy to the argument of lower annealing temperatures being responsible for faceting, we argue that higher binding energy for CoPd compared to AgAu induces a ligament faceting during electrolytic coarsening in a similar way. Indeed, when considering the rate equations for surface diffusion and dissolution in eqs 1 and 2, one finds that a higher/lower annealing temperature formally causes the same change in the rate constants as would be expected for a lower/higher binding energy. In a simple microscopic picture, a higher temperature promotes atomic movement processes in the same manner as a weaker binding to neighboring atoms does. Different annealing temperatures are equivalent to different binding energies, and thus different starting alloys for dealloying. Our results confirm the applicability of Erlebacher’s dealloying model^16^ to other material systems beyond AgAu. Furthermore, our results suggest that it is possible to obtain the same microstructure and even the same dealloying kinetics for AgAu and for CoPd at an elevated temperature.

### Dealloying *versus* Electrolytic Coarsening

Dealloying and coarsening per se are distinctively different processes. Dealloying, as the formation of a nanoporous structure from solid alloy, increases the surface area over time. Coarsening, on the other hand, with the growth of nanoporous ligaments and pores, decreases the surface area over time. Despite the converse effect of dealloying and coarsening on the overall surfaces area, both processes crucially depend on the same microscopic process of surface diffusion. While the process of electrolytic coarsening has been addressed in the previous section, here, we focus on the dealloying step solely.

Small-angle X-ray scattering in transmission geometry yields a signal that averages over all sample parts, from the newly formed porous layer right at the dealloying front to late-stage coarsening. Using the small-angle X-ray scattering technique in GISAXS geometry, a constant volume defined by the interaction volume of the incident X-rays with the sample is examined. Depending on the material system, the penetration depth of X-rays in the sample in GISAXS geometry is in the order of 60 nm (AgAu) and 130 nm (CoPd), which allow the quasi-local investigation of the dealloying process in that limited volume and thus a time-resolved study of both dealloying and electrolyte coarsening.

**Figure 5:**
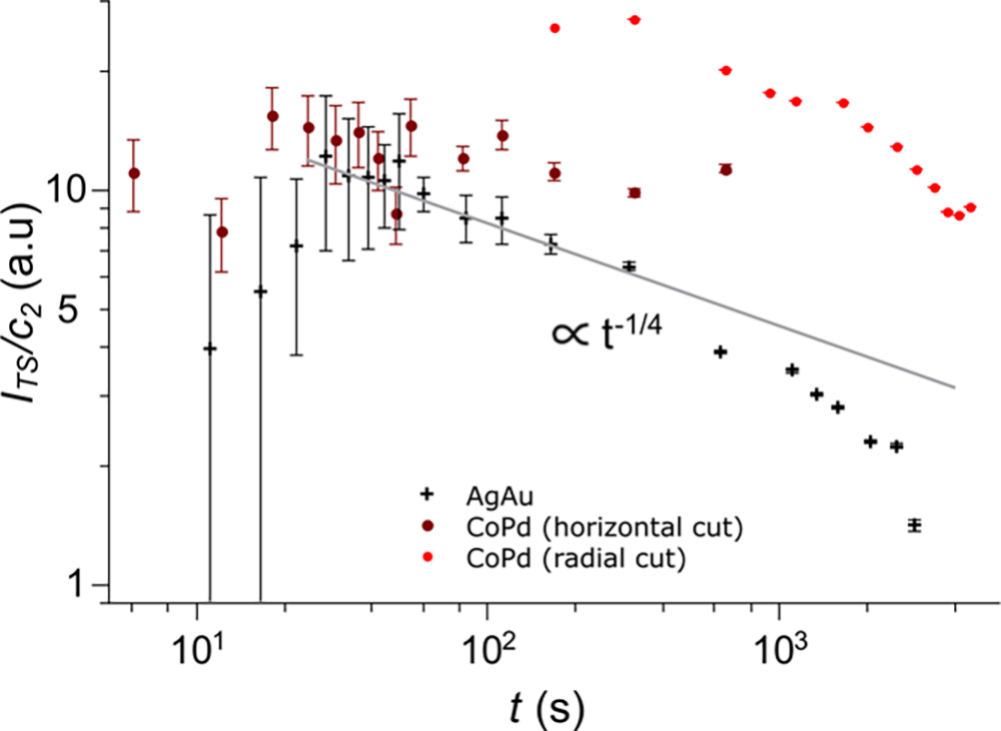
Evolution of surface area during dealloying. Temporal evolution of the ratio between scattering forwarded probability *I*_TS_ and the parameter *c*_2_ retrieved from fitting the measured data sets with the Teubner–Strey model.^40^ Data points for AgAu (CoPd) are shown as black crosses (red circles). For CoPd, both radial and horizontal cuts of the GISAXS patterns were fitted. The black line represents a decreasing surface area following a *t*^–1/4^ law.

Here, we introduce *I*_TS_/*c*_2_ as a measure of specific surface area (in the dimension of m^–1^), which is computed from the scattering forward probability *I*_TS_ and the fit parameter *c*_2_ from the Teubner–Strey model fit to GISAXS scattering data (see the Materials and Methods Section). The value *I*_TS_/*c*_2_ is presented as a function of time *t* for Au and Pd in Figure 5, while the equivalence of the results retrieved from fitting the dataset with the Choi–Chen and Teubner–Strey models is shown in Figure S7.

Two different dealloying regimes for AgAu are clearly visible in Figure 5 (black crosses). After an initial increase of surface area for the first 20–30 s, the surface area decreases over time for the remaining data points, roughly following a *t*^–1/4^ law as indicated by the black line. We assign the initial surface area increase to the true dealloying stage in the sampled volume, i.e., the conversion of solid alloy to nanoporous metal. This conversion occurs at a sharp phase boundary between alloy and nanoporous structure, which is referred to as the dealloying front (see Figure S8). Based on the interaction depth of X-rays in the experiment of about 60 nm and the period of increasing surface area, which marks the dealloying time, a velocity of the dealloying front can be estimated to *v*_D_ = 2–3 nm/s. In view of a decreasing dealloying front velocity with decreasing etching agent concentration for AgAu in free corrosion experiments,^23^ such a value appears reasonable considering the dilute acid concentration of 0.1 M here. A faster dealloying process with higher corrosion potentials can be expected for electrochemical dealloying compared to free corrosion, which is a direct result of the stronger driving force for dissolution imposed via the applied potential. This directly relates to the results for CoPd in Figure 5, where no period of increasing surface area can be observed for the data points in red. As the corrosion potential was effectively higher for CoPd, as shown in the Materials and Methods Section, a faster dealloying can be expected, which occurs prior to the first data point for CoPd in Figure 5. A linear relation between current density and etch front velocity has been suggested for electrochemical dealloying.^23^ Using this relation, a higher dealloying front velocity for CoPd is confirmed by the initial etching currents, which were about 5 times larger for CoPd compared to AgAu (not shown). Over the whole time span, the surface area for CoPd decreases, with a small jump apparent at the transition of GISAXS to SAXS due to intrinsic scaling differences in the two underlying models. No unambiguous power law behavior can be identified for the coarsening-related surface area decrease for the dealloying of CoPd.

Despite equal outer dimensions, the total etching process for CoPd takes a longer time of 4500 s compared to 1100 s for AgAu. While the dealloying step itself is faster for CoPd, as shown in Figure 5, the longer total etching time is related to a longer phase of dissolution from the already porous structure behind the dealloying front. This dissolution phase concurs with the observed coarsening stages for AgAu and CoPd in Figure 5. The preceding formation of nanoporosity in the dealloying process is governed by dissolution kinetics and thus faster for CoPd, which is observed as a faster dealloying front in Figure 5.

Trends in surface area extracted from the Teubner–Strey model in Figure 5 can also be compared to the ligament size parameters from the Choi–Chen model in Figure 4 exploiting the inverse proportionality between specific surface area and ligament size. Related to our measurements, an inverse behavior of *D*_p_ in Figure 4 and surface area *I*_p_/*c*_2_ in Figure 5 upon electrolytic coarsening (i.e., after 20–30 s for AgAu and right from the start for CoPd) can therefore be expected, which is confirmed by the extracted values from the experiment.

## Summary and Conclusions

In this study, we investigated the dealloying and electrolytic coarsening behavior of AgAu and CoPd alloys using *in situ* grazing-incidence small-angle diffraction supported with a combination of scanning transmission electron microscopy and kinetic Monte Carlo simulations. Results from different techniques were compared in reciprocal space, utilizing fast Fourier transforms of the STEM images and calculated scattering patterns from the KMC simulation. Scattering curves were fitted using the Choi–Chen model, where length scales of the nanoporous structures were extracted as a fit parameter. A discussion of dealloying kinetics was conducted on the basis of ligament–ligament distance as the characteristic length for nanoporous metals upon coarsening. While the kinetic behavior for AgAu confirms previous literature findings, a slower coarsening kinetics for CoPd was detected. We argue that dealloying of both alloys follows the same surface-diffusion-driven coarsening mechanism, with slower kinetics being a natural result of surface faceting due to the higher binding energy of CoPd.

## Supporting Information Available

Additional information on the experimental results and details about the conducted analysis (PDF)
