# Supplementary material for: In Situ Study of Nanoporosity Evolution during Dealloying AgAu and CoPd by Grazing-Incidence Small-Angle X-ray Scattering
Source: J Phys Chem C Nanomater Interfaces. 2022 Feb 17;126(8):4037–47. doi: 10.1021/acs.jpcc.1c09592 (PMC8900123; doi:10.1021/acs.jpcc.1c09592)
Supplement: Supplementary file 1 — jp1c09592_si_001.pdf [file jp1c09592_si_001.pdf]

Supporting information to the manuscript entitled

„**An *in situ* Study of Nanoporosity Evolution during Dealloying AgAu and CoPd by Grazing-Incidence Small-Angle X-ray Scattering** " submitted to Journal of Physical Chemistry.

Markus Gößler[1], Elisabeth Hengge[1], Marco Bogar[2,3], Mihaela Albu[4], Daniel Knez[5], Heinz Amenitsch[3*], and Roland Würschum[1*]

[1]*Institute of Materials Physics, Graz University of Technology, Petersgasse 16, 8010 Graz, Austria*

[2]*CERIC-ERIC C/o Elettra Sincrotrone, S.S. 14 Km 163.5, 34149 Trieste, Italy*

[3]*Institute for Inorganic Chemistry, Graz University of Technology, Stremayrgasse 9, 8010 Graz, Austria*

[4]*Graz Centre for Electron Microscopy, Steyrergasse 17, 8010 Graz, Austria*

[5]*Institute of Electron Microscopy and Nanoanalysis, Graz University of Technology, Steyrergasse 17, 8010 Graz, Austria*

Crystal structure of the alloys

X-ray diffraction (XRD) patterns were measured in a $\theta/2\theta$ geometry using a Bruker D8 Advance diffractometer. The device used a Cu anode (K_α: λ=0.154 nm) as the X-ray source. The range between 2θ=30° and 2θ=100° was investigated for the alloys before etching in the pristine state. The software DIFFRAC.EVA and a PDF (powder diffraction file) database were used for data evaluation and indexing. Crystal structures of both AgAu (Fig. S1) and CoPd (Fig. S2) alloys were identified as face-centered-cubic (fcc).

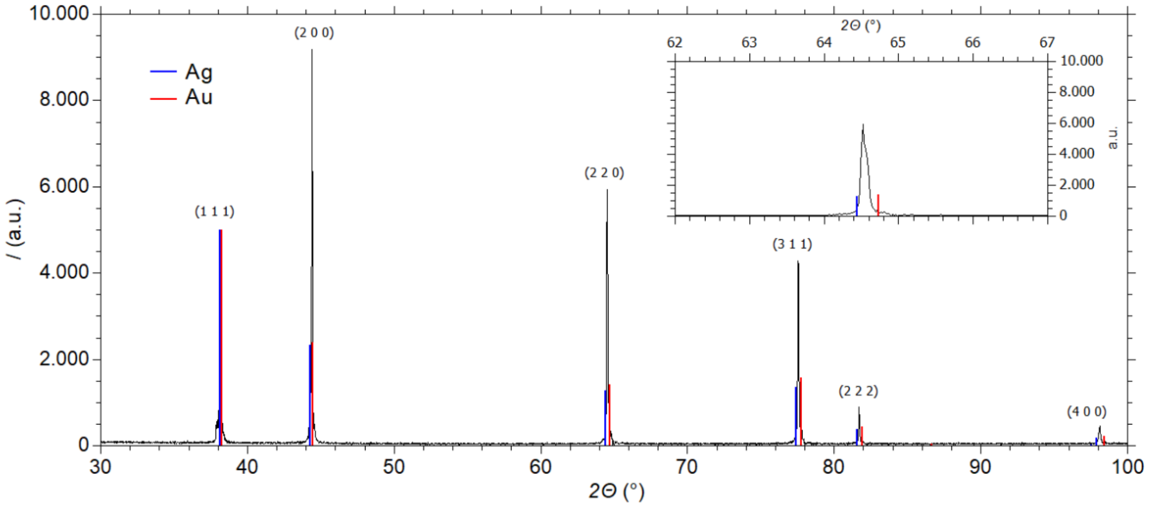

Fig. S1. X-ray diffraction (XRD) patterns of the AgAu alloy. Reference lines for Ag (blue – PDF 03-0931) and Au (red – PDF 01-1174) from the PDF database are drawn in the plot. The inset shows a magnification of the (220) peak.

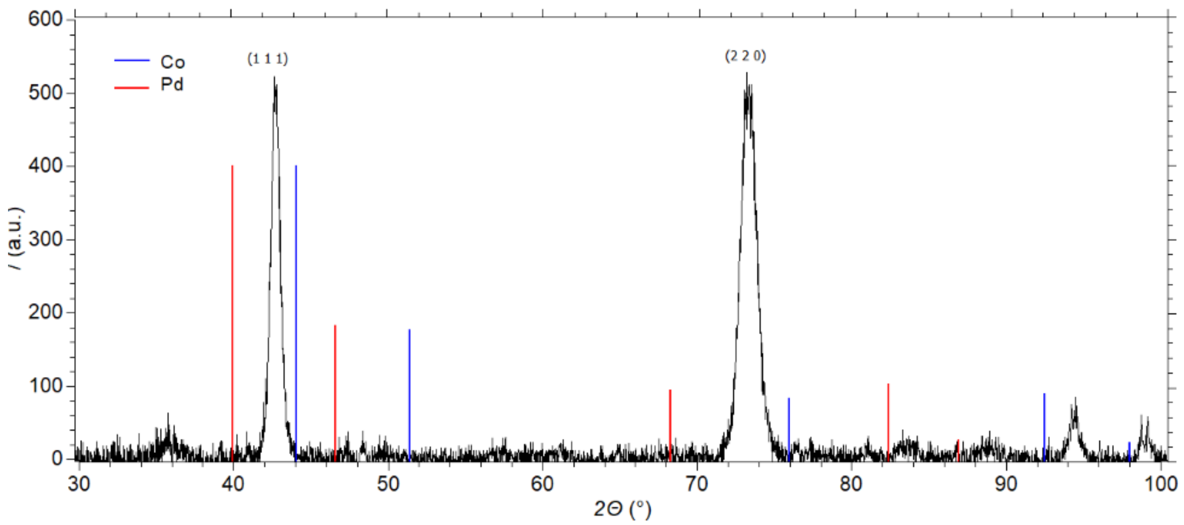

Fig. S2. X-ray diffraction (XRD) patterns of the CoPd alloy. Reference lines for Co (blue – PDF 88-2325) and Pd (red – PDF 88-2335) from the PDF database are drawn in the plot.

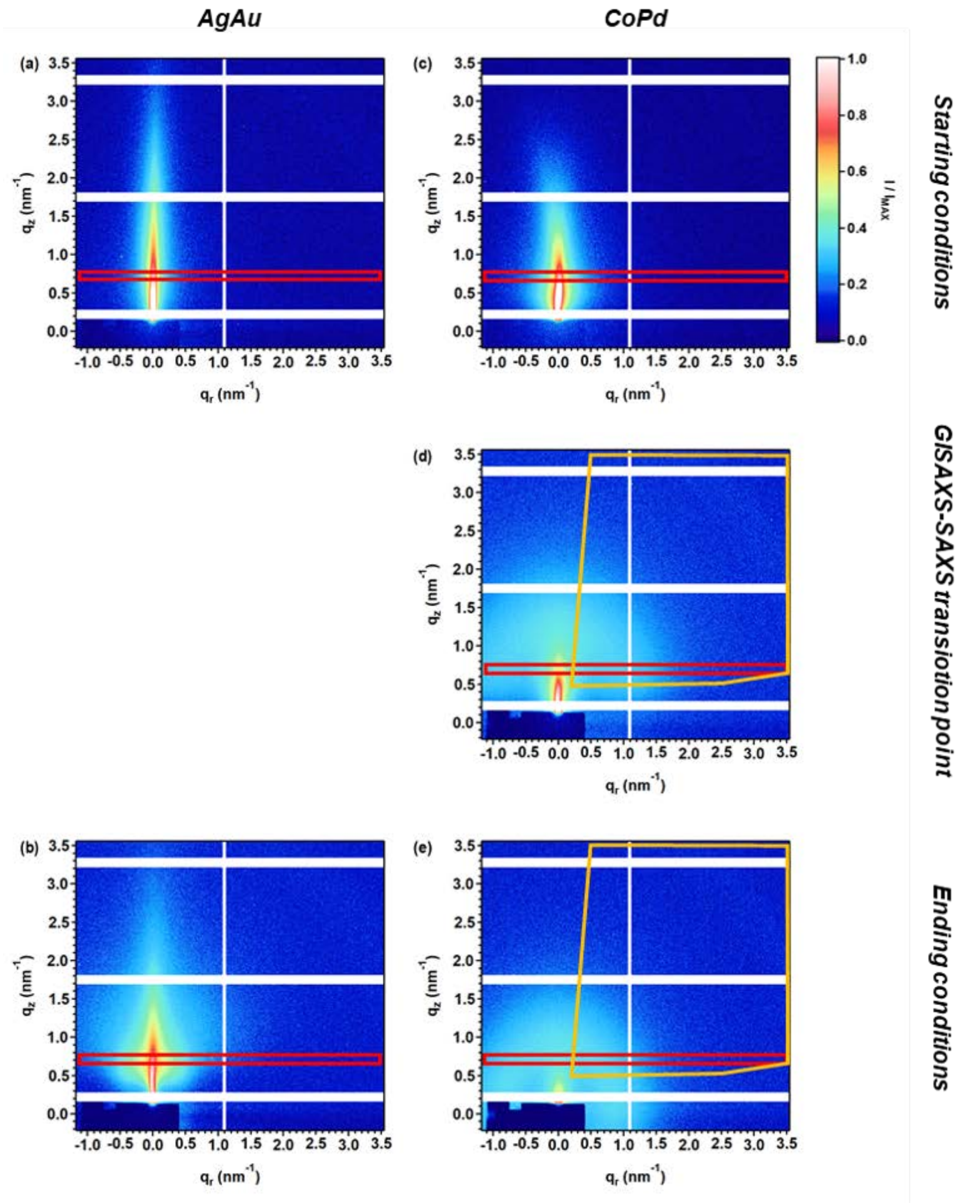

Fig. S3. GISAXS integration. 2D scattering patterns recorded from: (a, b) AgAu and (c, d, e) CoPd at different etching times: (a, c) at starting conditions, (d) at the transition point from GISAXS to SAXS analysis (for CoPd sample only) and (b, e) at ending conditions. The horizontal cut (in-plane direction) used for GISAXS analysis was obtained by integrating the scattering pattern within the red square. The radial cut used for transmission SAXS analysis was obtained by integrating the scattering pattern within the area defined by the orange lines.

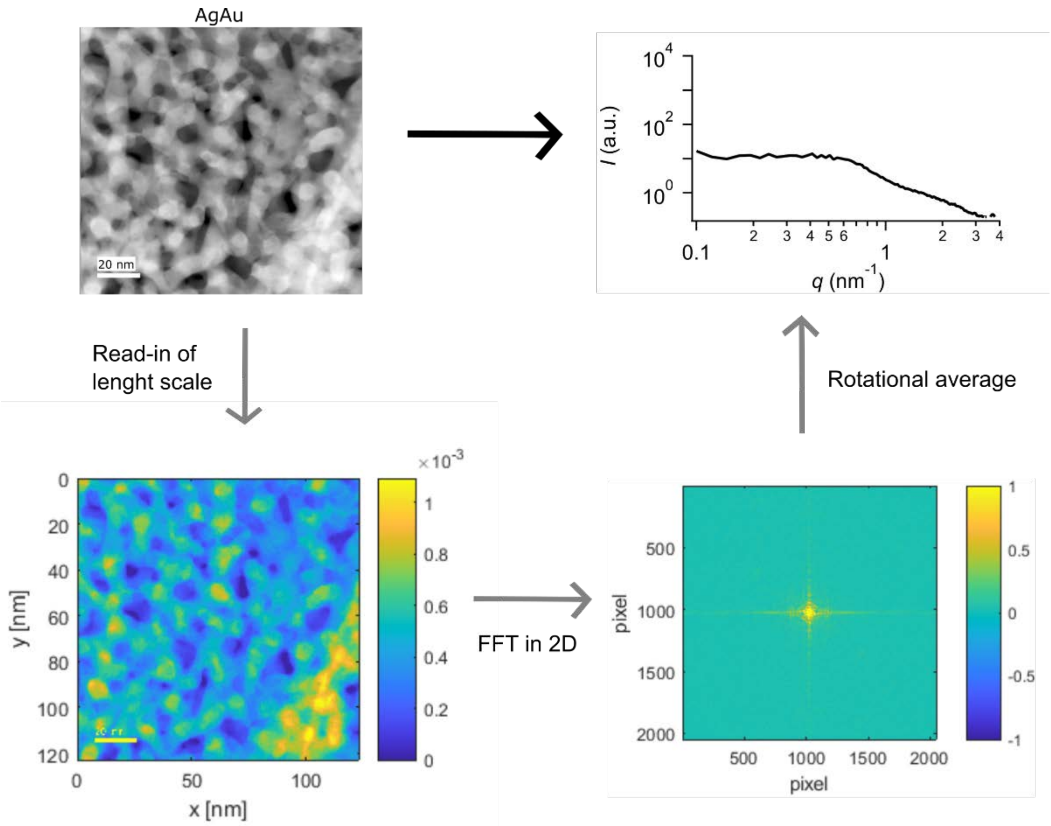

Fig. S4. Calculating 2D Fast Fourier Transforms from STEM images. Grayscale HAADF STEM images were assigned a length in nm per pixel. 2-dimensional Fast Fourier Transforms were then generated using the fft2 routine in MATLAB. Final calculated scattering patterns were obtained after rotationally averaging over the the 2D-FFT image and converting the pixel scale back into wavenumbers q, using the defined length per pixel.

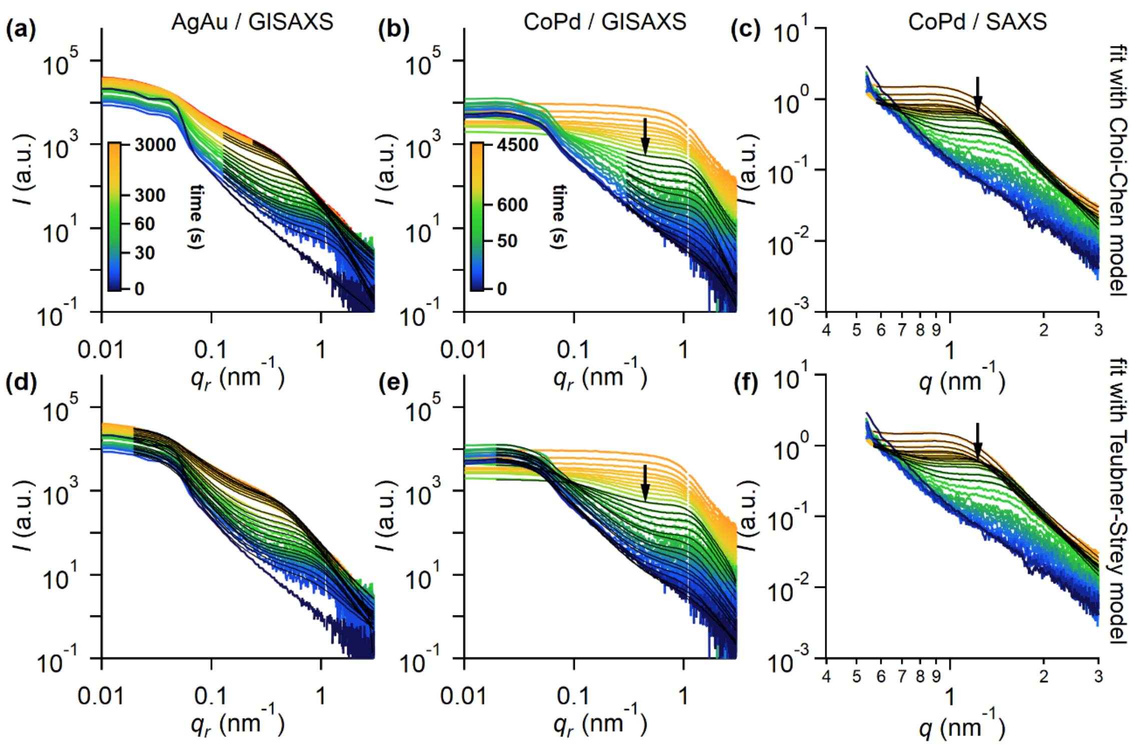

Fig. S5. Fitting results for *in situ* GISAXS. Time resolved GISAXS scattering pattern and the respective fitting results using the Choi Chen (upper row) and the Teubner-Strey (lower row) model. Results for AgAu are shown in (a) and (d). For CoPd, in (b) and (e) the horizontal cuts (GISAXS geometry) and in (c) and (f) the radial cuts (SAXS geometry) are shown. The point of transition (655 s) is marked by the black arrow.

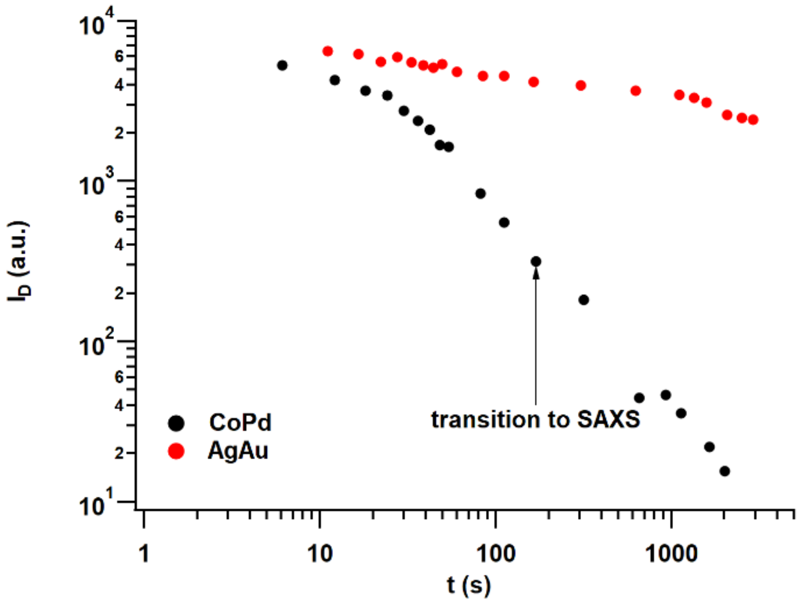

Fig. S6. Diffused scattering comparison. Temporal evolution of the scaling factor of the Guinier-Porod model, I_D, retrieved by fitting the GISAXS scattering patterns with the Teubner-Strey model.

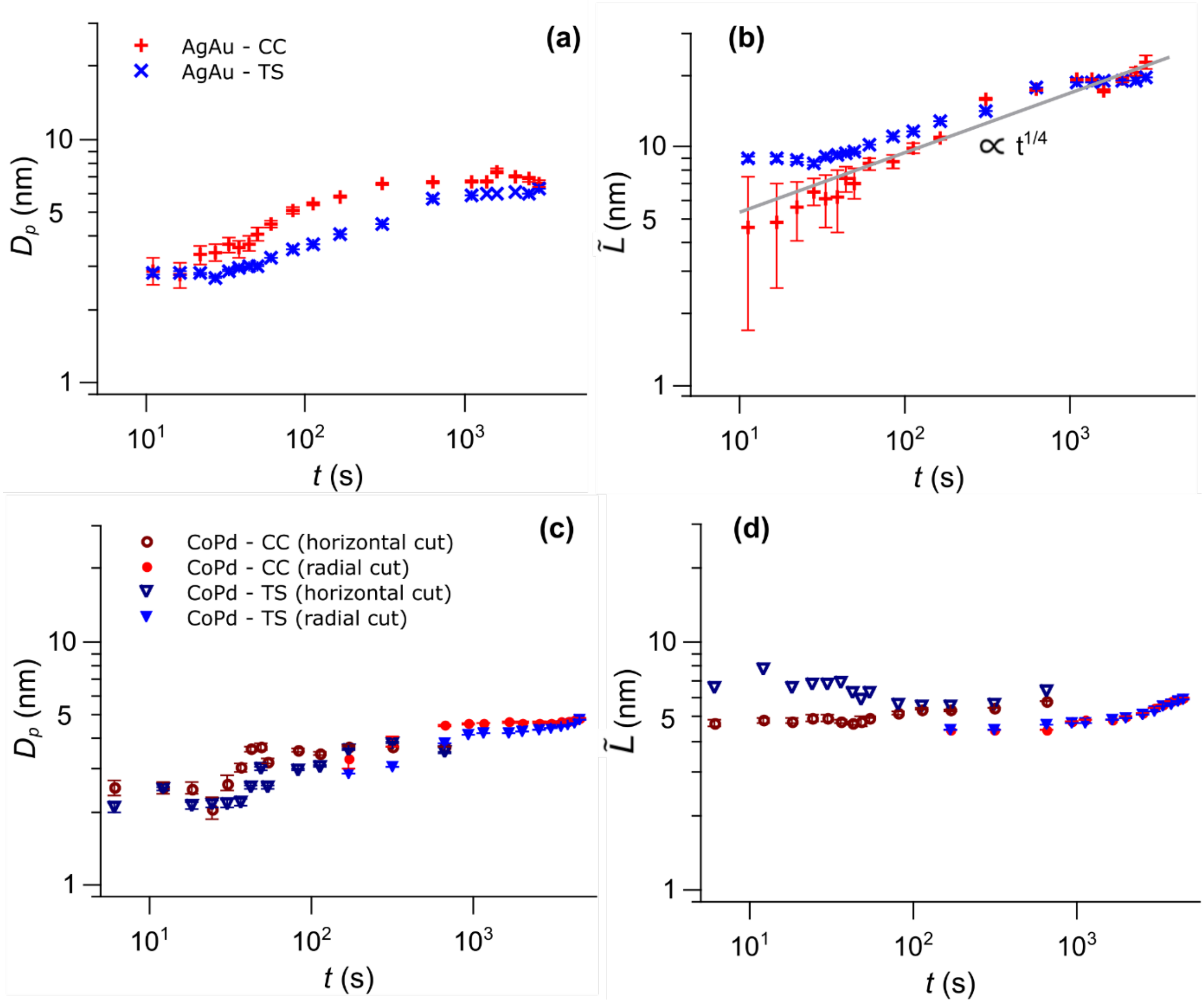

Fig. S7. Comparison of structural sizes during dealloying from different models. Pore size D_P and interpore spacing $\tilde{L}$ as a function of etching time t from the experiment for AgAu (a and b) and for CoPd (c and d) in double-logarithmic representation. Data points from the Choi and Chen model which are also given in the main text in Fig.4, are shown in Black, while data points extracted from fits according to the Teubner-Strey model are shown in blue. Size parameters are obtained via fitting horizontal cuts of the GISAXS patterns in Fig. 2. Fit parameters to different cut directions (horizontal, i.e. along the Yoneda, and radial, i.e. average over all directions – SAXS geometry) are shown for CoPd in c and d. The continuous grey lines in (b) correspond to a slope 0.25, which is characteristic for surface diffusion driven coarsening (d~ $t^{1/4}$). Differences in fit parameters from the Choi-Chen and Teubner-Strey models arise mainly at lower times t, whereas there is decent agreement at later times.

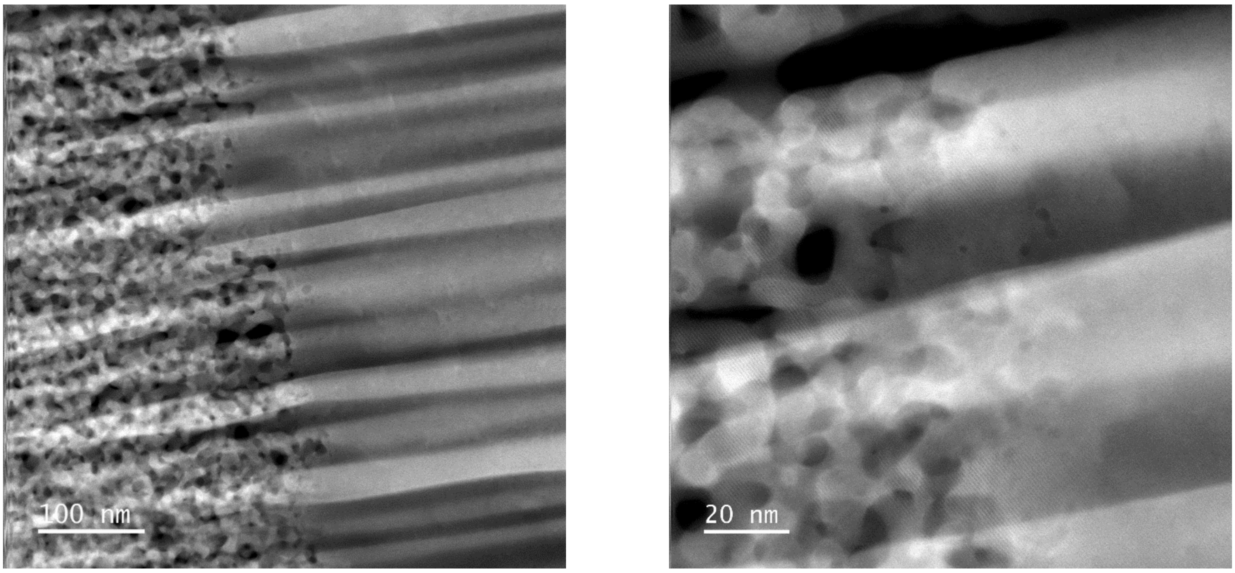

Figure S8. The dealloying front: STEM images of AgAu dealloyed for 60 s at different magnifications. For the dealloying conditions the reader is referred to the experimental section in the main text. The dealloying front is visible as a sharp transition between AgAu alloy (right part in both images) and already nanoporous Au (left part).
